# Stealthy Encroachment: Unraveling an Encounter With Renal Myelolipoma

**DOI:** 10.7759/cureus.58628

**Published:** 2024-04-20

**Authors:** Shakti Sagar, K.M. Hiwale, Pravin Gadkari, Suhit Naseri, Simran Khan, Miheer M Jagtap

**Affiliations:** 1 Pathology, Jawaharlal Nehru Medical College, Datta Meghe Institute of Higher Education and Research, Wardha, IND

**Keywords:** kidney, glomerular filtration rate, hematopoietic elements, flank pain, extra-adrenal myelolipoma

## Abstract

Myelolipoma of the kidney is an exceedingly unusual benign tumor of hematological components mixed with mature adipose tissue. We present a case of a 59-year-old male who presented with left flank pain and was found to have an atrophic left kidney on imaging studies. A computed tomography (CT) scan revealed a small and shrunken left kidney with an extrarenal pelvis. A diethylenetriamine pentaacetate (DTPA) scan results showed a total glomerular filtration rate (GFR) of 45.6 ml/min with a non-functional left kidney. The patient underwent a left nephrectomy, and a histopathological examination confirmed the diagnosis and highlighted the distinctive morphological features of this rare entity. Postoperatively, the patient experienced a complete resolution of symptoms. This case underscores the importance of considering myelolipoma in the differential diagnosis of renal masses and highlights the successful management of symptomatic cases through surgical intervention. Awareness of this rare tumor is crucial for accurate diagnosis and appropriate management. Further studies are needed to elucidate the natural history and optimal treatment strategies for renal myelolipomas.

## Introduction

Kidney myelolipoma is a relatively uncommon benign tumor that can mimic other renal tumors. Most physicians may be ignorant of this entity. Mature adipose tissue and mature hematopoietic components are combined in different ratios to form it. The adrenal gland is the most frequently involved location. According to autopsy data, its frequency in extra-adrenal locations ranges from 0.08% to 0.4%, which is extremely unusual [1]. Rarely have a few cases of extra-adrenal myelolipomas in the kidney been documented; instead, they are more commonly observed in the retroperitoneum, mediastinum, stomach, liver, thorax, pelvis, presacral region, and even the thyroid glands [2]. It can be very difficult and requires specialized knowledge to misdiagnose uncommon entities like myelolipoma. Its favored location, the adrenal gland, is where the first occurrence was reported by Gierke in 1905 [3]. There is no correlation with gender, although the condition is more common in the seventh decade of life. Usually, myelolipomas are discovered by accident. Treatment is not necessary for myelolipomas that are asymptomatic and non-hemorrhagic. In general, the patient has a great prognosis in most cases. Large masses, however, have the potential to bleed and induce necrosis. Myelolipoma can present with non-specific symptoms such as flank pain, hematuria, or palpable abdominal masses in some cases. In our case, the patient presented with left flank pain with elevated creatinine.

## Case presentation

**Figure 1:**
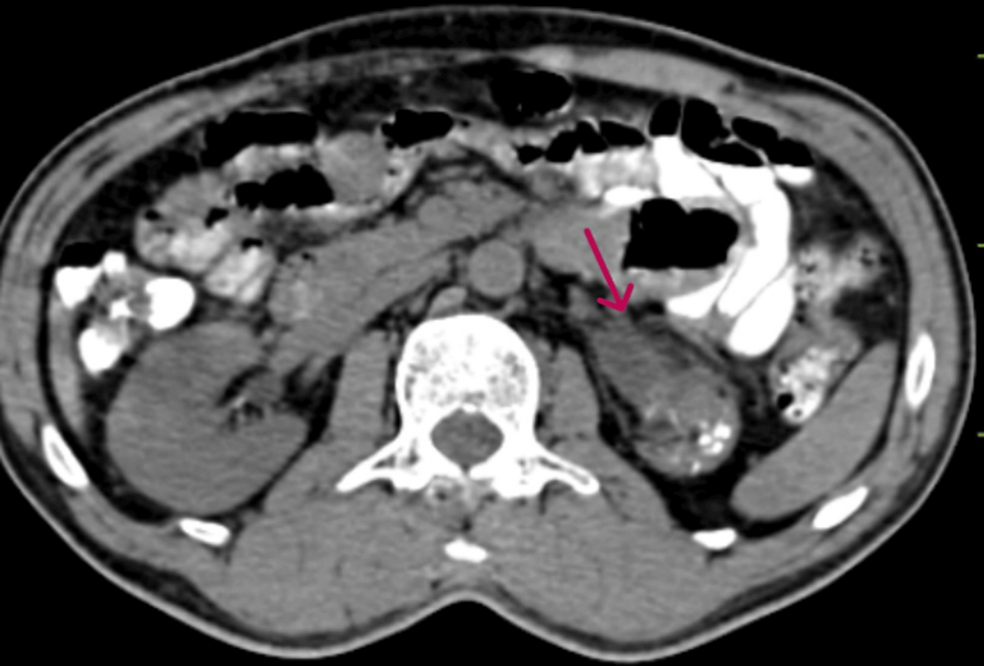
A contrast-enhanced CT of the abdomen and pelvis of the patient shows a small, shrunken, and atrophic left kidney with marked thinning of the cortex and distorted parenchyma. The extrarenal pelvis is seen on the left side.

A 59-year-old male came to urology OPD and presented with left flank pain that had persisted for the past three months. Nothing unusual, including lymphadenopathy or hepatosplenomegaly, was found during a physical examination. Laboratory investigations indicated leucocytosis and elevated creatinine levels. Diethylenetriamine pentaacetate (DTPA) scan results were as follows: glomerular filtration rate (GFR) was 45.6 ml/min/1.73 m2 with a non-functioning left kidney. Radiological imaging, including contrast-enhanced computed tomography (CECT) of the abdomen and pelvis, revealed the left kidney measuring 6.5 x 3.5 cm, small and shrunken in size, with marked thinning of the cortex and distorted parenchyma (Figure 1).

**Figure 2:**
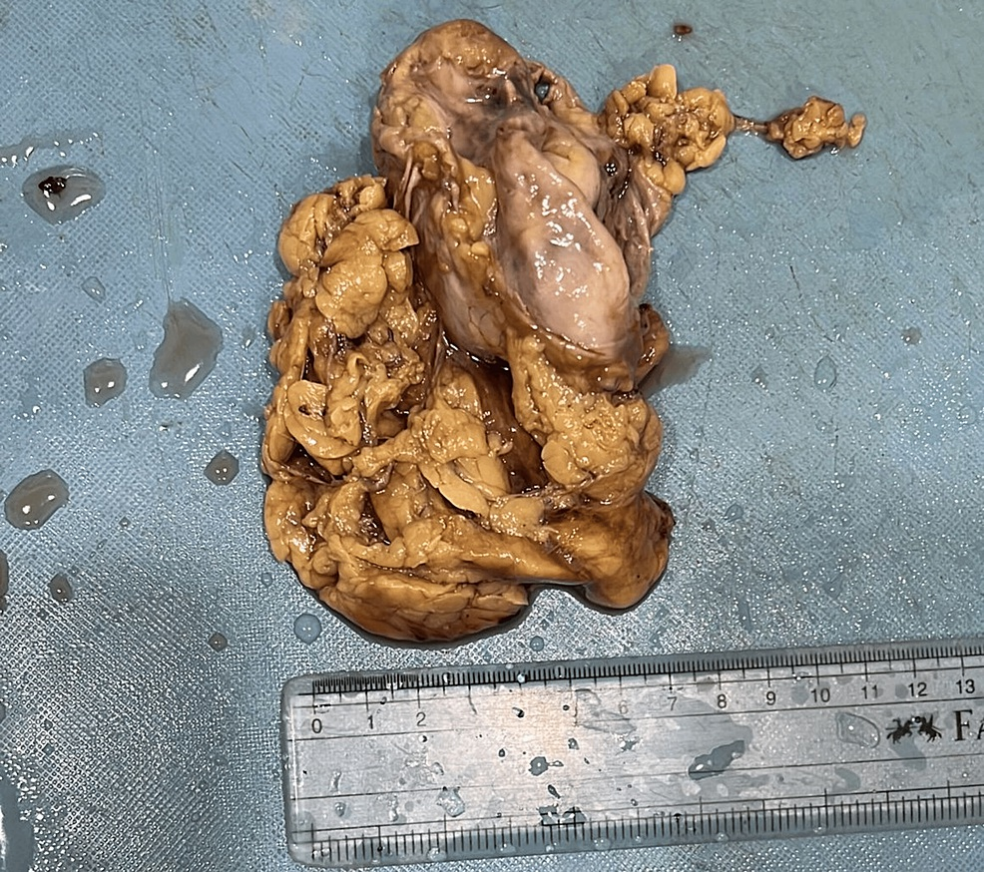
Gross photograph of the left nephrectomy specimen

The extrarenal pelvis is seen on the left side. There are marked cortical and parenchymal calcifications noted on the left side. Minimal perinephric fat stranding. The right kidney is normal in size, shape, and axis. Following a left nephrectomy, the specimen was sent for histopathological analysis. On gross examination, the left kidney measured 5.5 x 3.5 x 1.8 cm with perinephric fat attached (Figure 2).

**Figure 3:**
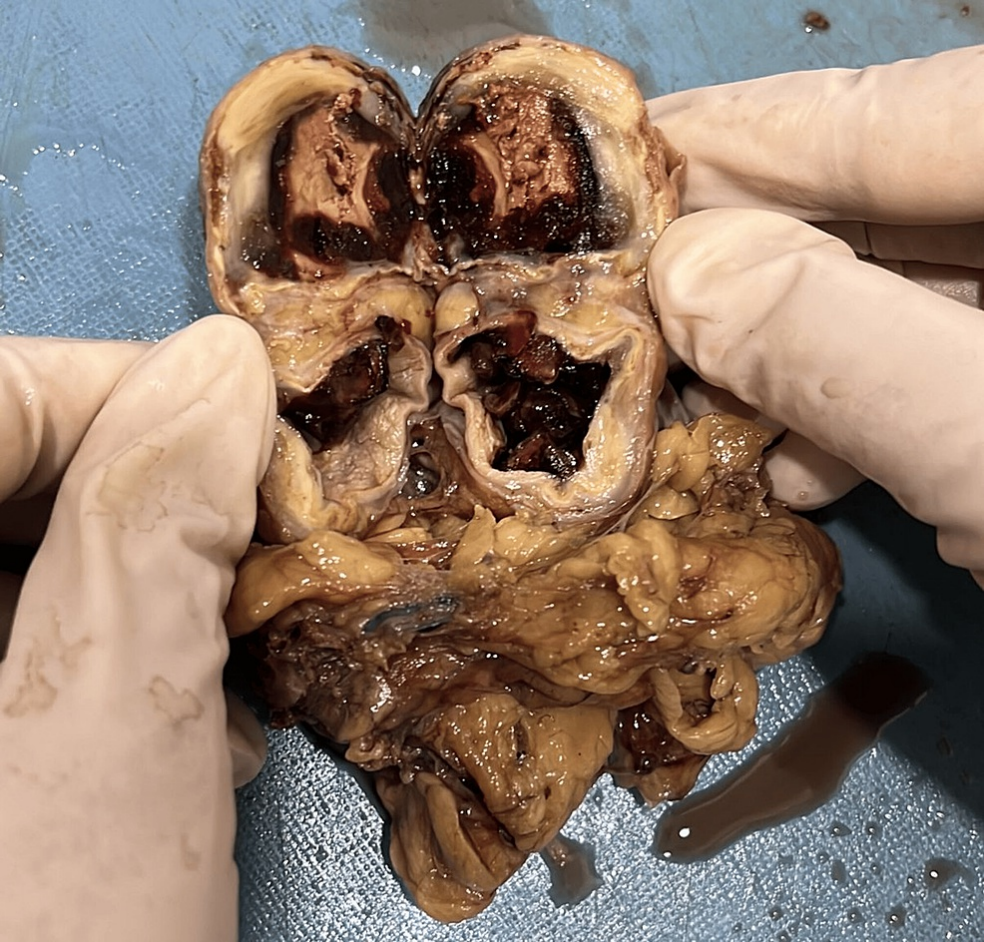
In the cut section, a well-circumscribed, yellowish-white tumor was identified in the upper pole of the left kidney. There is also dilation of the calyces and a thinned-out cortex.

In the cut section, there is a loss of corticomedullary differentiation and hemorrhagic areas identified in the medulla. There is dilation of the calyces and a thinned-out cortex. A well-circumscribed, yellowish-white tumor was identified in the upper pole of the left kidney, measuring 1.3 x 1 cm (Figure 3).

**Figure 4:**
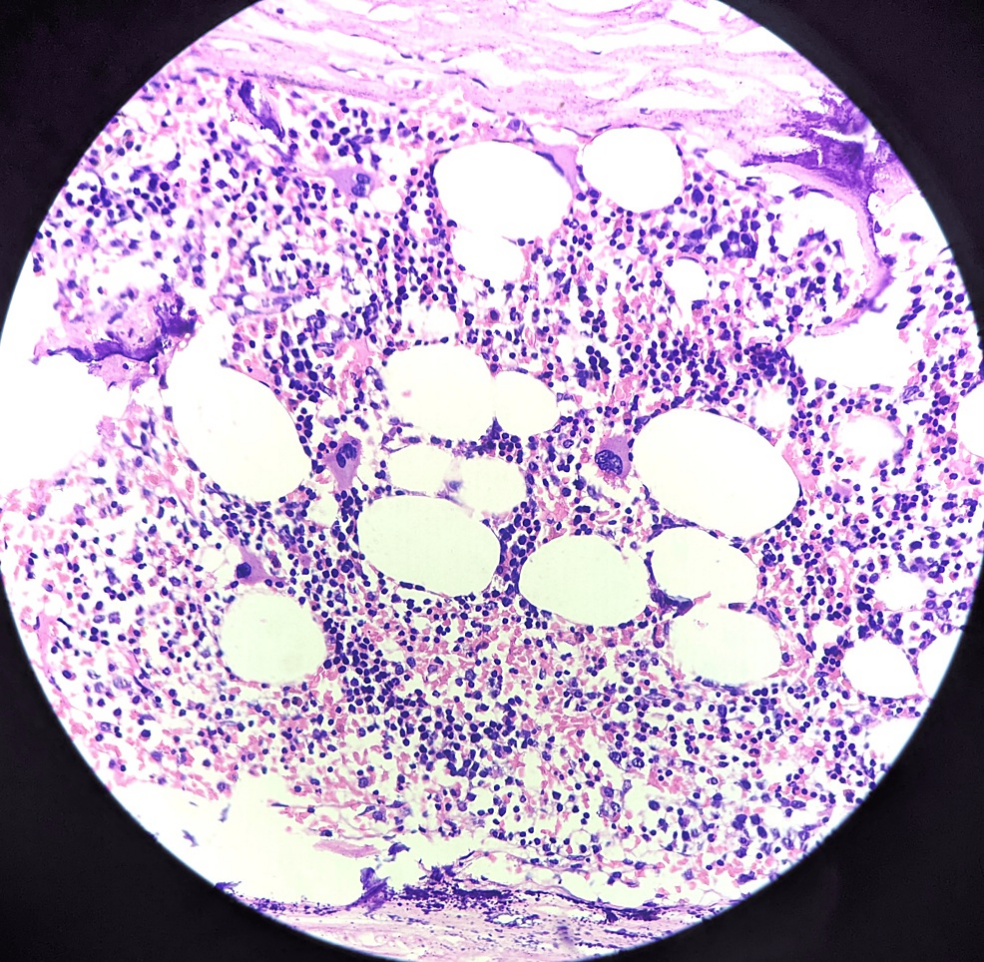
Microscopy showed bone marrow hematopoietic elements and mature adipocytes (40x magnification). Haematoxylin and Eosin (H&E) stain was used

**Figure 5:**
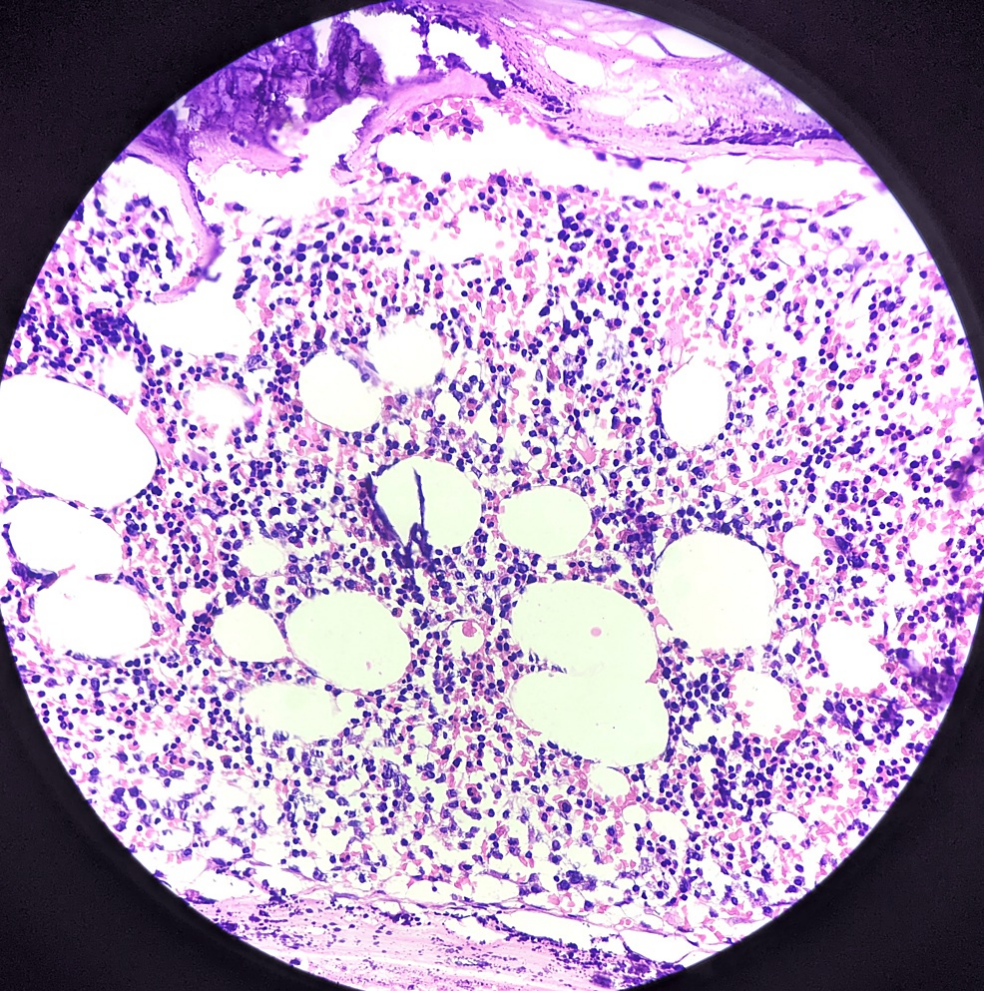
Microscopy also showed undergoing necrosis ensheath by capsule with foci of dystrophic calcification (40x magnification). Haematoxylin and Eosin(H&E) stain was used

Microscopic examination of the specimen showed bone marrow hematopoietic elements and mature adipocytes (Figure 4) undergoing necrosis ensheath by capsule with foci of dystrophic calcification beneath the affected residual kidney (Figure 5).

Histopathological features were suggestive of myelolipoma of the left kidney. No adrenal rest was found. At the three-month follow-up, the patient's surgical course was uneventful, and he is still clear of illness. Six months after the operation, surveillance imaging revealed neither distant metastatic disease nor local recurrence, and he was asymptomatic.

## Discussion

When compared to other organs, the adrenal gland is frequently the site of benign mesenchymal neoplasms called myelolipomas, which are composed of adipose tissue and hematopoietic components [4]. Charles Oberling ultimately gave the name myelolipoma to this tumor in the adrenal gland in 1929, although Edgar von Gierke initially reported it in 1905 [5]. It's unclear exactly how myelolipoma develops. While one hypothesis stated that it is a kidney tumor origin, another idea revealed clonal cytogenetic aberration and proposed that it is hamartoma or clusters of choristomatous hematopoietic stem cells that transmigrated into other places during fetal life [6].

Elderly people are more likely to develop adrenal and extra-adrenal myelolipomas. Most of the time, they go undetected during a radiological scan for another ailment and are frequently clinically quiet. Abdominal or flank discomfort is the most prevalent complaint among symptomatic individuals. Although there are no clear-cut radiological standards for identifying extra-adrenal myelolipoma, these formations can appear in several places. Fortunately, the imaging features of adrenal myelolipoma can be used to detect these abnormalities [7].

Radiologic techniques, such as CT scans, are useful in properly diagnosing gland myelolipoma since they comprise mature adipose tissue. However, since they might be mistaken for other tumors, extra-adrenal and fat-poor myelolipomas are more challenging to identify before surgery.

Grossly, myelolipoma appears as a solid, well-defined, unencapsulated tumor in the kidney. The sliced surface of a myelolipoma often displays patches of irregularly distributed brownish-frizzle tissue mixed with soft yellow fatty tissue. Under the microscope, a combination of normal hematopoietic cells from all three hematopoietic cell lineages (megakaryocytic, erythrocytic, and granulocytes) and adipose tissue may be seen in myelolipoma.

A fat-containing retroperitoneal tumor might have several differential diagnoses, including retroperitoneal liposarcoma, adrenal or extra-adrenal myelolipoma, angiomyolipoma, and retroperitoneal teratoma. According to Venyo [8], there have only been less than 50 cases of extra-adrenal myelolipoma documented, and there have only been 30 occurrences of myelolipoma related to or situated in the kidney.

One of the most prevalent benign kidney lesions is angiomyolipoma [9]. Under the microscope, a typical angiomyolipoma is often a triphasic tumor with variable percentages of mature adipose tissue, spindle, and epithelioid smooth muscle cells, and dysplastic blood vessels. Angiomyolipoma with a predominance of fat imitates other diseases like liposarcoma and myelolipoma. Retroperitoneal liposarcoma frequently has no symptoms until the tumor grows to a large size.

Reactive extramedullary hematopoietic tumors, which typically develop in conjunction with myeloproliferative diseases or chronic hemolytic anemia, are also taken into account in the differential diagnosis. Extramedullary hematopoietic tumors are characterized by erythroid hyperplasia and a preponderance of hematopoietic components under the microscope. Fat does not have a larger role in the procedure [10].

Because of their minimal risk for bleeding and quick development, tiny asymptomatic lesions are often treated conservatively with radiological follow-up. Surgical intervention is necessary for symptomatic individuals, who have an expanding tumor or have an unclear diagnosis. In our case, myelolipoma of the kidney is confirmed by histopathological analysis. The long-term prognosis is excellent. After six months, it was recommended to have a follow-up CT scan, which showed no local recurrence, and he was asymptomatic.

## Conclusions

In conclusion, renal myelolipoma is typically benign and has a favorable prognosis. Careful consideration should be given to factors such as tumor size, symptoms, and growth patterns when determining the optimal management strategies. Multidisciplinary collaboration between urologists, radiologists, and pathologists is crucial for the appropriate diagnosis, appropriate treatment planning, and long-term follow-up of these patients. Further research is warranted to elucidate the underlying pathogenesis of renal myelolipoma and to refine management algorithms, particularly for asymptomatic cases. Additionally, long-term studies are needed to assess the recurrence rates and outcomes associated with different treatment modalities. Overall, our experience with this case contributes to the growing body of literature on renal myelolipoma and emphasizes the importance of vigilance in diagnosing and managing this rare renal tumor.
